# “It’s like these CHCs don’t exist, are they featured anywhere?”: Social network analysis of community health committees in a rural and urban setting in Kenya

**DOI:** 10.1371/journal.pone.0220836

**Published:** 2019-08-08

**Authors:** Robinson Njoroge Karuga, Maryse Kok, Patrick Mbindyo, Femke Hilverda, Lilian Otiso, Daniel Kavoo, Jaqueline Broerse, Marjolein Dieleman

**Affiliations:** 1 LVCT Health, Nairobi, Kenya; 2 Athena Institute, Vrije Universiteit, Amsterdam, The Netherlands; 3 Royal Tropical Institute (KIT), Amsterdam, The Netherlands; 4 Jomo Kenyatta University of Agriculture and Technology, Juja, Kenya; 5 School of Health Policy and Management, Erasmus University, Rotterdam, The Netherlands; 6 Community Health and Development Unit, Ministry of Health, Nairobi, Kenya; 7 Amsterdam Public Health Research Institute, Amsterdam, The Netherlands; World Health Organization, SWITZERLAND

## Abstract

**Background:**

In Kenya, Community Health Committees (CHC) were established to enhance community participation in health services. Their role is to provide leadership, oversight in delivery of community health services, promote social accountability and mobilize resources for community health. CHCs form social networks with other actors, with whom they exchange health information for decision-making and accountability. This case study aimed to explore the structure of a rural and an urban CHC network and to analyze how health-related information flowed in these networks. Understanding the pathways of information in community settings may provide recommendations for strategies to improve the role and functioning of CHCs.

**Methods:**

In 2017, we conducted 4 focus group discussions with 27 community discussants and 10 semi-structured interviews with health professionals in a rural area and an urban slum. Using social network analysis, we determined the structure of their social networks and how health related information flowed in these networks.

**Results:**

Both CHCs were composed of respected persons nominated by their communities. Each social network had 12 actors that represented both community and government institutions. CHCs were not central actors in the exchange of health-related information. Health workers, community health volunteers and local Chiefs in the urban slum often passed information between the different groups of actors, while CHCs hardly did this. Therefore, CHCs had little control over the flow of health-related information. Although CHC members were respected persons who served in multiple roles within their communities, this did not enhance their centrality. It emerged that CHCs were often left out in the flow of health-related information and decision-making, which led to demotivation. Community health volunteers were more involved by other actors such as health managers and non-governmental organizations as a conduit for health-related information.

**Conclusion:**

Social network analysis demonstrated how CHCs played a peripheral role in the flow of health-related information. Their perception of being left out of the information flow led to demotivation, which hampered their ability to facilitate community participation in community health services; hence challenging effective participation through CHCs.

## Background

Since the Alma-Ata declaration of 1978, many low- and middle-income countries (LMICs) started to acknowledge that community participation in health is a key ingredient in building responsive, sustainable and effective health systems. Community members have to be empowered to take part in decision making during the design, implementation and evaluation of health programs [1]. Empowerment is a process and outcome that involves “creating opportunities for those without power to gain knowledge, skills and the confidence to take decisions that affect their lives (page 1)” [2]. Communities are empowered to participate in making decisions related to health services when they have the information and capacity to identify and prioritize their health needs, and then develop strategies to address these needs [3]. Community-based committees are one way of empowering communities to participate in decision making in health matters and are officially recognized bodies operating within health systems in many LMICs [4].

Over the last twenty years, a number of LMICs began reforming their health systems and set up community-based committees that were envisioned to provide an interface between communities and health care providers; these committees go by different names, namely primary health care committees, village health committees or community health committees, among others. Members of these committees were expected to provide leadership in holding front-line health workers accountable, providing feedback to health workers, and conducting planning, implementation and evaluation of community-based health services [5–7]. One such reform was the institutionalization of community-based committees that would facilitate participation of community members in decision-making related to community-based health interventions and services [8, 9]. Depending on their context, these community-based committees perform different roles. For example, committees may be involved in mobilizing households to participate in health promotion campaigns while at the same time, they hold primary health care providers accountable for delivery of health care, encourage uptake of primary health care and mobilize resources for community health by lobbying local governments [10]. A number of studies have documented gaps between national health policies and actual operationalization of these committees as mechanisms for community participation. For example, studies conducted in India showed that members of several Village Health, Sanitation and Nutrition Committees were not aware of their roles, had not been trained and had never participated in the development of local health plans [11, 12]. Other studies in Nigeria showed that despite their official role in facilitating community participation in health services, Community Health Committees did not effectively link with the wider health sector. There were also instances where key actors, including committee members, did not clearly understand the roles of these committees [10, 12, 13].

### Community health strategy in Kenya

In efforts to enhance access to equitable and affordable health care in Kenya, the Ministry of Health launched the Community Health Strategy in 2006. In this strategy, the Ministry of Health recognized communities as the foundation of the health system and defined communities as: “specific groups of people, usually living in a defined geographical area, who share common values, norms, culture and customs, and are arranged in a social structure according to relationships which the community has collectively developed over a period of time” [14, 15]. This strategy specifies that communities should be empowered to participate in the delivery of health care services. Community units are the operational structures for the delivery of community health services and their geographical boundaries were defined according to government administrative units, referred to as sub-locations, which cover a population of approximately 5,000 people. Each community unit is then linked to primary health care facility (a dispensary or health center). Community health services (health education, health promotion, follow-up, and referral) in each community unit are delivered by Community Health Volunteers (CHVs) and are supervised by Community Health Extension Workers (CHEWs), who are professional public health staff employed by the government to coordinate community health services in each community unit. Delivery of community health services in each community unit is overseen by a Community Health Committee (CHC).

CHCs are one of the two types of community-based health committees in Kenya. The other community-based health committee is referred to as the Health Facility Management Committee, which is mandated to oversee the delivery of facility-based health services [16]. Health Facility Management Committees were established in 1998 and consist of local leaders, health facility staff and lay community members [17]. CHCs were established in 2006 as the first structure to be constituted in a community unit, before selection of CHVs. CHC members are usually influential persons and leaders such as village elders, women group leaders and youth leaders. Community members nominate 11 to 13 CHC members during public gatherings referred to as barazas (monthly community level meetings convened by local chiefs). According to the community health strategy, the chair of a CHC should be a community member–who is then nominated to be part of the Health Facility Management Committee. Each CHC should have a maximum of two CHVs, one of whom is the treasurer. The CHEW is the secretary and technical advisor to the CHC [14].

CHCs are expected to provide leadership and oversight in the implementation of community health services within the community unit. More specifically, CHCs are expected to oversee the work done by CHVs, plan, coordinate and mobilize community members for monthly dialogue days and quarterly action days, develop community unit annual health work plans, mobilize resources for implementing community health activities and collaborate with Health Facility Management Committees on issues concerning the local health facilities [14, 18, 19].

### Social networks of community health committees

In order to move beyond community participation in health as tokenism and allow CHCs to play their role in health systems they require access to health-related information, to other actors and to resources. CHCs could be seen as operating in a social network because of their relations and interactions with other actors. Lunsford et al. (2015) demonstrated how social networks can be utilized using the Community Health System Strengthening Model. This model leverages on existing social networks and structures to improve health outcomes at community level. In their publication, Lunsford *et al*. documented how Health Extension Workers and community teams (composed of village and religious leaders) in Ethiopia utilized social networks at community level to disseminate health messages, increase uptake of antenatal care and HIV testing among pregnant women and improve sanitation and hygiene. The same model was applied in Tanzania where community groups and committees used their existing networks to create demand for HIV testing, deliver health promotion messages and improve retention of community members in HIV care clinics [20].

Understanding how CHCs operate in their social networks, involves mapping the different actors, their positions as well as how these actors influence the role of CHCs. For instance, interactions of CHCs with community members and health workers involves exchanging information that may create opportunities for mobilizing resources within the network [21]. Attributes like trust among individuals may influence reciprocity and cooperation among members of a social network [22]. There has been limited empirical research on social networks of CHCs. Our research aimed to assess the social networks of community units in a rural area and an urban slum. We were in particularly interested in the position of CHCs in their networks and how health related information flowed among actors.

## Methods

We studied social networks of CHCs- as entities—in a rural and urban slum using a case study design. This design facilitated an in-depth analysis of relationships and health-related information exchange [23]. Data were collected between May and November 2017.

We conducted a Social Network Analysis using mixed methods. Social network analysis is a method used for providing insights into relationships between groups, people and institutions [24]. This approach has been applied for describing, exploring and understanding relational aspects of health [25]. By assigning numerical values, “1” when there is a relationship and “0” when there is none, social network analysis enables us to describe networks and to characterize the position of actors in their networks [26]. Examples of network characteristics are: density, degree of centrality, reciprocity, diameter of the network and betweenness; social network analysis also enables us to visualise social networks using sociograms [27]. We utilized social network analysis to concurrently visualize the number of connections that facilitate exchange of health-related information between actors in a network, the nature of these connections (reciprocal or one sided) and the position of actors in the network [28]. In our case, actors represented organizations and formal groups. Table 1 illustrates a comprehensive summary of network characteristics that we measured in this study.

**Table 1:** Definitions of characteristics of the social network examined in this study [26, 29, 30].

Characteristics in the social network	Operational definition
Relational ties	Ties connect actors within a network. Presence of a connection is indicated by Yes (value = 1) or No (value = 0). A tie is not only considered as presence of a connection, the direction of the tie is also taken into account. Therefore, reciprocal connections are counted as 2 ties, while one sided connections are counted as 1 tie.
Density	The purpose of measuring network density is to give a sense of how well information flows among actors in the CHCs’ networks. We measured density by dividing the number of **actual** ties between actors in the network by the number of **potential** ties that would have existed. Density therefore ranges between 0 and 1. A fully connected network has a density value of 1. This means that every actor is exchanging information with every other actor.
Reciprocity of the whole network	We measured reciprocity by dividing the number of **actual** reciprocal **ties** between actors in the network by the number of **all ties in the network.** The larger this percentage, the more provide-and-receive relations between actors exist. The purpose of this measurement is to show that information is not only shared one-way from one actor to another but mutually between actors in the network.
Centrality	This measurement indicates how central to the flow of health-related information an actor is within the overall social network. We derived the **degree of centrality** by dividing the number of other actors with whom a specific actor exchanged health-related information by the total number of actors in the network. We defined two specific measures of degree of centrality, namely **in-degree** and **out-degree**. **In-degree** refers to the number of actors that give health-related information towards an actor. **Out-degree** is the number of actors that receive health-related information from an actor. We measured **betweenness centrality** to determine the number of times an actor was a “bridge” for health-related information flow between other actors who are not directly connected to each other, along the shortest path.
Diameter	The diameter of a network represents the size of the social network. This measurement informs us how quickly health related information flows within the network and how integrated the different actors in the network are. To calculate the diameter of a network, we first determine the shortest distance between every pair of actors. The longest of these paths is the diameter of the network. Related to the diameter is the **average path length**, which represents the total of the shortest paths between all actor pairs, divided by the total number of pairs. Average path length shows the average number of steps it takes for health related information to flow from one actor to another.

### Study locations

We conducted this study in Kajiado and Nairobi Counties. Kajiado County is a rural region that has a population of 687,321 people and the Maasai are the predominant ethnic community. Livestock rearing is the major economic activity followed by small-scale agriculture. Nairobi County is the Kenyan capital with an estimated population of four million people. Nairobi County is highly cosmopolitan and has diverse socioeconomic and cultural settings. Community health services are delivered in the peripheral urban slums. The majority of residents in Nairobi’s slums work as casual laborers and in running micro-businesses. Community units in both counties were selected in consultation with health managers at county level. Each of the study locations had a government owned primary health care facility (Health Center) that provided services such as skilled delivery, immunizations and growth monitoring. CHVs and CHEWs referred community members to these facilities for primary health care. Criteria for selecting community units for the study were: having an active CHC that met on a regular basis and community sites that were active in mobilizing communities for quarterly dialogue days.

### Data collection and sampling

Focus group discussions (FGDs) and semi-structured interviews were carried out to provide data on the actors and their relational ties in the social networks. We also gathered complementary qualitative data on the roles of different actors in sharing health-related information in both social networks (S1 Table). These case studies were conducted as part of a broader study that was investigating the contextual factors that influence CHCs as they play their roles in community participation. Our FGD and interview topic guides were informed by a conceptual framework on contextual factors that influence health committees in developing countries [31]. The topics that were explored during FGDs and interviews focused on how health administration, community, society-wide reforms and health workers influenced CHCs.

**Focus group discussions:** We conducted FGDs with CHCs and community members. Community members in both study sites represented women, men, youth, micro-business owners, farmers (in the rural setting) and religious leaders.

FGDs with CHCs explored their relational ties with other actors in the community unit and the type of health-related information that they exchanged. We did this by asking them to recall all actors that they exchanged health-related information with on a regular basis. We examined the information exchanged by the CHC as an entity or by individuals in their role as CHC members rather than the information exchanged by individual CHC members in their other community roles. FGDs with community members discussed their perceptions on the roles of the CHCs in overseeing community health services.

All CHC members were eligible to participate in the FGDs and community members had to have lived in the community unit for at least one year to be eligible to participate in the FGDs. CHEWs supported us in organizing FGDs by scheduling them in private rooms within the compound of the local health facilities and requesting participants to volunteer to take part in the FGDs. The primary researcher (RK) moderated the FGDs and a research assistant supported with note taking. In the urban slum setting, FGDs with the CHC and community members took 28 minutes and 43 minutes, respectively while in the rural settings, FGDs with CHC and community members took 57 minutes and 33 minutes, respectively.

**Semi structured interviews:** We conducted semi-structured interviews with health care professionals (CHEWs, nursing officers in charge of local health facilities, sub-county level government health managers). These participants represented the health system institutions where they worked. The semi-structured interviews explored the relational ties that these health professionals had in the community units and how health-related information flowed in these units. We asked these health providers to recall and list all the actors in the community unit and reporting structures that they regularly exchanged health information with. Using topic guides, we asked the following questions about each actor that they listed: the kind of health information they exchanged, frequency of information exchange, how they shared health information and why it was important to share this information with the respective actors. We conducted the interviews in private rooms within the health facilities for approximately 30 minutes each.

**Sampling in the rural community:** In the rural community unit, we purposively sampled 17 participants based on the roles they played in delivery of community health services. Two FGDs were organized; One was composed of six CHC members (3 Male, 3 Female) and the second FGD included seven community members (5 Female and 2 Male). Community members who participated in FGDs were purposively sampled with support from the CHEW. The four health professionals were interviewed in the rural community unit: the sub-county level government health managers, CHEW and nursing officer in charge of the link health facility.

**Sampling in the urban slum:** In the urban setting 16 people participated. We conducted two FGDs; one with four CHCs members (2 Male and 2 Female) and one with five community members (3 Female and 2 Male). FGD participants in the urban slum were also purposively sampled with support from the CHEW. We then interviewed seven health professionals: three CHEWs, the nursing officer in charge of the local health facility, sub-county level government health managers.

### Data management and analysis

Social network analysis was conducted using UCINET 6.632 software [32]. This software was used to visualize the networks in sociograms and to compute the network measures described in Table 1. To avoid losing data from actors who could not participate, they were retained in the network as long as they were mentioned by another actor. A team of four trained research assistants transcribed all audio recordings into MS Word 2013. Transcripts were then transferred into Nvivo 10 for coding [33]. We analyzed qualitative data using the thematic analysis approach[34]. Themes in our analytical framework were based on a conceptual framework developed by George *et al*. This conceptual framework defines four overlapping contextual factors that influence health committees. These factors are community, health facilities, health administration, and society factors [31]. Despite our approach being deductive, we were open to adding emerging themes that would enhance our understanding on social networks of CHCs. The analysis framework was applied to code verbatim quotes into themes and sub-themes such as CHC factors, societal factors, health worker perceptions of CHCs, information flow among others. The analysis process entailed familiarizing ourselves with the data, coding into the analytical framework, charting data into a framework matrix and interpreting data in themes [35, 36]. We used narratives from the FGDs and interviews to support the findings on information flow within the networks and complement quantitative findings on the social network analysis.

### Ethics approval

Clearance to conduct this study was obtained from the Kenya Medical Research Institute Ethics Review Committee (NON-SSC PROTOCOL NO.144). Administrative clearance to conduct the study was granted by the Kajiado and Nairobi County Health Departments.

## Results

### CHC characteristics

CHC members in both community settings had low levels of formal education, with all of them having attained basic (primary and high school) education. CHC members were also leaders in other sectors within the community such as security committees, local school boards and community projects. By virtue of their leadership roles in the community, CHC members were trusted by others. As one CHC member put it during an FGD:

*“…CHCs are not only limited to working on health matters we are involved in other ways*. *For instance*, *I also work at the chief’s office*, *like this mzee* [elder] *is in charge of nyumba kumi* [community policing initiative], *I mean we have to be people who are trusted in the community”* (Male participant, rural CHC FGD)

Notably, most of the CHC members also served as CHVs. For example, half (6 of 12) of the CHC members in the rural setting served as CHVs. They reported being in these double roles because: a) they were recognized more when they worked as CHVs since county government and NGOs preferred working with CHVs than with the CHCs, and b) CHVs received trainings from NGOs and county governments–these opportunities were not available for CHC members who did not also serve as CHVs. CHEWs in both settings informally appointed these CHVs as “senior CHVs”. This created a new informal cadre of peer CHV supervisors who supervised fellow CHVs. CHEWs were still the primary supervisors in the community unit. Characteristics of both CHCs and community settings are summarized in Table 2.

**Table 2:** Characteristics of CHC in the rural and urban community unit.

	Description of the rural CHC and community unit	Description of the urban slum CHC and community unit
Population served by the CHC	Approximately 7,600	Approximately 5,400
Number of Villages	6	6
Estimated number of households in their jurisdiction	1,627 households	1,858 households
Number of CHVs who actively provided community health services	28	25
Mean age of CHC members	48.2 years	40.8 years
Gender		
Male	6	5
Female	6	10
Literacy level	All members had basic literacy skills	All members had basic literacy skills
Occupation of active CHC members	All reported to be small scale farmers	All stated that they were small business owners
Length of service for current CHC	At least 4 of the 6 members had been in the CHC for at least 5 years	7 years
Number of CHC members who regularly participated in CHC activities	6 out of 12 CHC members	5 out of 11
Community representation in the CHCs	Six CHVs, Five Former Traditional, Birth Attendants (TBAs), One Head of Community Policing	Three CHVs, One community leader, One youth leader, Three women leaders in the community, One Person Living with HIV
Number of members trained in CHC roles	One member (Former Chairperson who is still a member)	One member who is the chairperson and is also the village elder
Representation by CHC members in other committees within community	Community policing, committee, water committee, schools board of management, village elders council	Peace committee, community elders, community policing and schools board of management

### Information flow between CHC and direct contacts in the network

CHCs in both sites shared information with the CHEWs about health care needs from the community, such as interventions needed from the health system to address communicable disease outbreaks. CHCs requested CHEWs for health promotion commodities such as deworming drugs and water purification tablets. CHEWs then relayed this information to either the officers in charge of the health facility or the sub-county level government health managers. CHEWs were also central in receiving feedback on the quality of health services in the local health facilities from CHCs who represented the community. Both CHCs relayed community members’ complaints about health care in the local facility through CHEWs. Examples of complaints from community members were negative attitudes of health providers during service provision and absenteeism by staff in the health facilities. CHEWs then relayed these complaints to the nurses in charge of the local health facilities. Nurses in charge of both local health facilities also relayed health-related feedback to the community through the CHEW, who then passed the information through the CHCs.

Chairs of both CHCs planned quarterly dialogue days together with CHEWs and local Chiefs. Together, they agreed on the agenda for the dialogue days. CHCs relied on the local Chiefs to address challenges they experienced while overseeing community health services. One example was when dealing with community members in religious sects that do not believe in immunizing their children as observed by a community member:

*“The committee members have also been helpful; there is a certain religious group here who don’t go to hospital*. *There was an outbreak and they joined hands with the CHEW*, *doctors and the office of the chief to take the children and give them immunization”* (Male participant, rural community members FGD)

Local Chiefs in both sites updated the CHC Chair on the latest government directives and their implementation. This information was exchanged during other meetings that the CHC Chairs were part of or during spontaneous face-face meetings. For example, Chairs of both CHCs were also members of their local community policing committees, which were chaired by local Chiefs. It was during these meetings that local Chiefs relayed information on government directives and policies such as directives against female genital mutilation, prevention of early marriage of girls, among others. CHCs would, in turn, inform the community policing committee of households that were defaulting health-related government policies and would enlist their support in enforcing them. CHC members would occasionally accompany the community policing committee to enforce the directives in the households as one member stated:

*“Like there is a time we had a measles outbreak*, *the child is not able to be brought to the hospital because of religious stands*, *we are forced to steal that child and bring her to hospital”* (Female participant, rural CHC FGD)

Since some CHC members in both study sites also served as informal peer supervisors of CHVs, they mainly exchanged information about reporting of data and mobilizing communities for dialogue days. CHVs relayed information from the community about their health needs through the CHC. In both sites, CHCs played an important role in motivating CHVs to continue serving in their voluntary roles as one CHEW stated:

*“CHCs have been able to unite those CHVs with the community at large because you know CHVs are volunteers and they are the ones who do a lot of work so the CHCs can motivate them by telling them to go on with that work*, *to work and they don’t leave”* (Female rural CHEW, SSI).

The type of health-related information that actors exchanged directly among themselves in both the urban slum and rural CHC social networks is summarized in Table 3.

**Table 3:** Summary of information that was directly exchanged between CHCs and four actors in both the rural and urban slum community units.

Information provided by:	Frequency of information sharing	Information provided by:
**CHC to CHEW**		**CHEW to CHC**
• Priority health needs from the community that require action • Community concerns about quality of care in the local health facility • Information about community members who had defaulted treatment and tracing them • Submission of monthly data collected by CHVs for entry into the Health Information Management System	*This information was often shared during monthly face-to-face meetings with those who were also CHV supervisors*. *Minutes were often kept by the CHEW*	• Feedback from health facility on health services • Feedback on uptake of health services • Updates on the quality of monthly reports collected by CHVs in the community
**CHC to Local Chief**		**Local Chief to CHC**
• Health-related agenda items that required to be discussed during the quarterly community dialogue day meetings • **Relevant to rural setting only**—Information about households that had defaulted government policies such as female genital mutilation, immunization of children under 5 years	*Most of this information was exchanged face-to-face during meetings with the Chief*. *Local Chiefs were ex officio members of CHCs and they also received information from CHC Chairpersons in informal discussions*, *beyond the scheduled meetings*	• Government directives related to health e.g. immunization of children, free maternity services • Follow up on action items agreed upon during dialogue days for implementation
**CHC to CHVs**		**CHVs to CHC**
• Emphasis on the importance of collecting correct community data and timely submission of monthly reports to the CHEW • Joint review of monthly household data between CHCs and CHVs • Joint planning of the quarterly dialogue days and strategies for mobilizing community members to participate • Motivation to continue serving in their voluntary role • Conflict resolution between CHVs and community members	*CHCs and CHVs met on a monthly basis to review reports collected in the community*. *The majority of the health information exchanges happened in these meetings*. *Both CHCs kept minutes of their meetings*. *A number of CHC members also acted as peer supervisors of CHVs*. *This enhanced communication of health information with other CHVs*	• Seeking support in resolving difficult situations encountered during service delivery in the households e.g. community members who do not comply with immunization, digging pit latrines etc. • Conflicts that have arisen with community members during service delivery
**CHC to Community policing committee (this is relevant for the rural CHC)**		**Community policing committee to CHC**
• Plans on how to enforce government directives in households that have defaulted	*Information exchanges mainly happened in face-face meetings*. *Most times during the community policing committee meetings*	• Alerts on households that defaulted on compulsory government health directives
**CHC to Community members**		**Community members to CHC**
• Importance of constructing and using latrines for sanitation and boiling drinking water • Emphasis to households in the community unit to seek skilled delivery services in local health center • Alert the community of any disease outbreaks in the community unit • In the rural community unit, discourage female genital mutilation and inform household that it is an offence	*CHCs shared information with households during the dialogue days*	• Complains of poor service in the local health center

A notable observation was that both CHCs did not have any form of connection with the committees responsible for overseeing management of the local health facilities. This is a departure from the policy guidelines, which stipulate how CHCs should be represented in Health Facility Management Committees.

*“Since then for about 2–3 years*, *I have never been called to be informed of any meeting* [of the Health Facility Management Committee]*; I’ve never heard”* (Male participant, urban slum CHC group discussion).

We also asked CHCs whether they exchanged health-related information with elected grass root level political leaders called Members of the County Assembly (MCAs). In the Kenyan context, MCAs are political leaders who are elected during the 5-year general elections to represent Ward level constituents in the county level parliament. MCAs’ legislative roles include policymaking, approval of county budgets and plans and holding the county government accountable. Both CHCs in the rural and urban setting did not exchange health information with MCAs, as was captured during the group discussion with the CHC in the urban slum:

**Moderator**: *Do you deal with politicians*?

**Male respondent**: *No*, *we don’t deal with politics*.

**Moderator**: *How about when you have proposals*?

**Male respondent**: *None; where can they be found*?

(Male respondent, urban slum CHC group discussion)

#### Structure of the social networks

**Fig 1:**
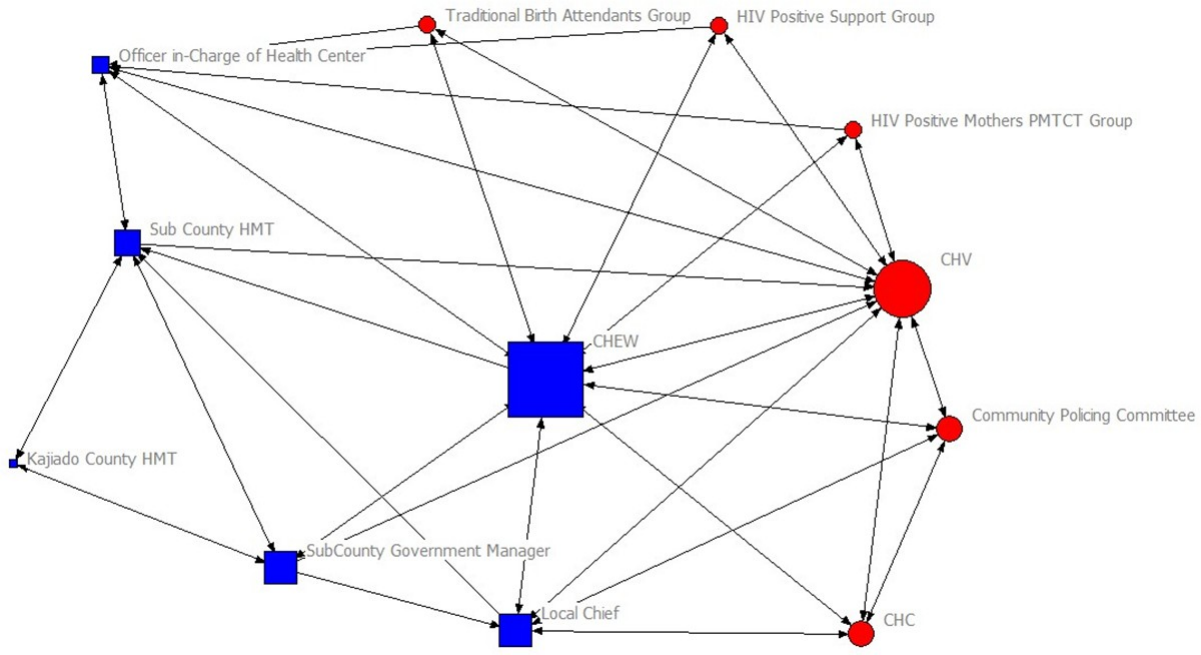
Social network for the rural CHC whose context is in a pastoral, rural community in Kajiado County. **Note:** Arrows in the sociograms indicate direction of the information flow. Reciprocal information exchange is indicated by the double arrows. Blue circles denote individuals who originate from the rural community and red boxes denote actors who represent government institutions. The size of the nodes corresponds to their degree of centrality.

**Fig 2:**
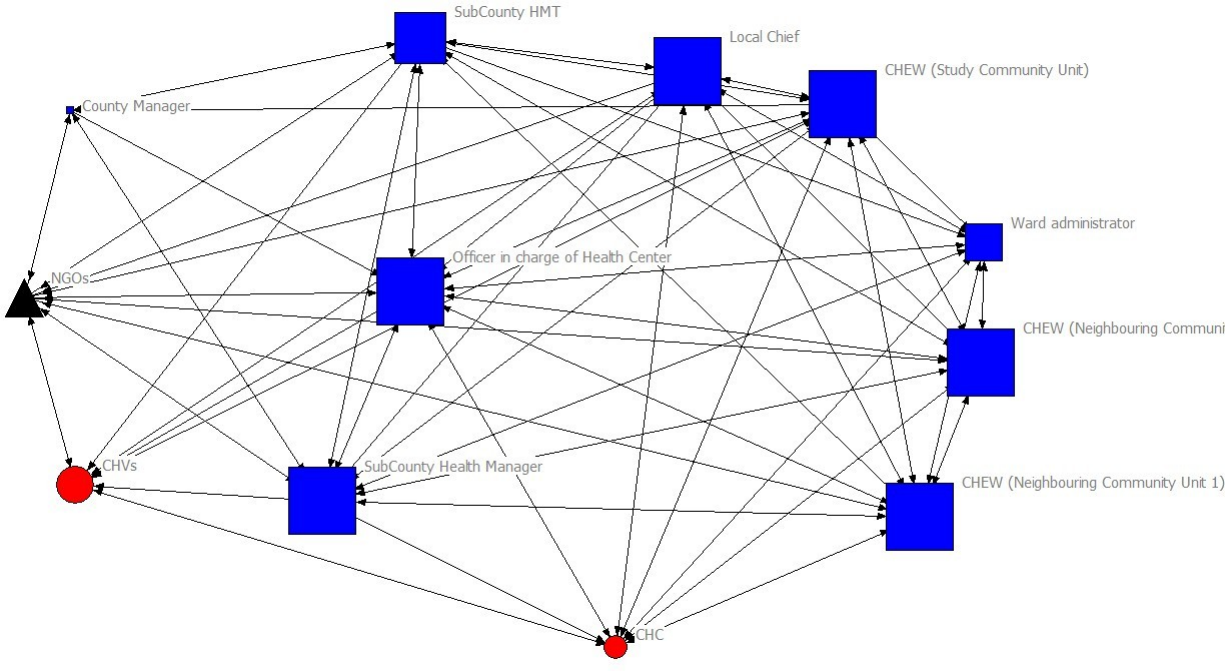
Social network for the urban slum CHC in Nairobi County. **Note:** Blue circles denote individuals who originate from the rural community and red boxes denote actors who represent government institutions. The size of the nodes corresponds to their degree of centrality. The triangle represents NGOs that implement health programs at community level.

The sociograms in Figs 1 and 2 visualize the connections between actors in the rural and urban CHC’s social networks, respectively. Information exchange is visualized by pointed lines connecting the actors. Each of the social networks had 12 actors, meaning that each had 132 potential relational ties and 66 possible reciprocal ties. The rural CHC network had 54 actual ties within the network, compared to 100 ties in the urban slum CHC. The rural CHC network had 46 reciprocal ties between actors, while the urban network had 92.

The rural CHC’s social network consisted of 12 actors. Six actors in this network originated from the community and the other six represented government departments. Out of the 12 actors in the urban slum CHC’s network, only two actors (CHVs and CHC) originated from the community. Nine actors represented government institutions and one group of actors was composed of non-government organizations that were implementing health projects. One difference between the rural and urban slum network was that the CHC in the urban slum exchanged information with CHEW from neighboring community units within the slum. This was because the CHC was located in a densely populated slum and there were blurred administrative boundaries. There was also high mobility of residents in this urban slum setting.

The rural CHC relied on their CHEW to act as a bridge when exchanging health information with the clinical officer in-charge of the local health facility, community support groups and the sub-county level managers. The CHC in the rural setting had a limited number of direct connections with other actors, namely, the CHEW, community-policing committee, CHVs and the local Chief, who were also members of the CHC. The CHC in the urban slum exchanged health-related information with other actors without relying on the CHEW to act as a bridge.

A notable observation was the number of individuals with double roles in both networks. For example, besides individuals having a double role as CHV and CHC member, local Chiefs were *ex-officio* members of the CHC and were also members of other community-based committees such as the peace committees, community policing committee, elders’ council and other community development committees (e.g. rural agriculture and water project committees). CHEWs were the secretaries to both CHCs. Membership of individuals with influential double roles in the CHC did not contribute to making the CHCs prominent in health information exchange within both networks, for example with the Health Facility Management Committee. This is observed by how peripheral CHCs were in the sociograms illustrated in Figs 1 and 2. At sub-county level, government health managers were also members of the Health Management Teams and were also the CHEWs’ direct supervisors.

The NGOs in the urban community unit had relational ties with all sub county level government health managers, frontline health providers and CHVs. They however did not have any relational ties with CHCs. First, NGOs had to inform the sub-county and local frontline health care workers about their intentions to implement community-level health projects. Second, these NGOs are involved in implementing health projects such as training CHVs and health workers in quality improvement and management of non-communicable diseases, among others. Information from NGOs reached CHCs indirectly through the CHEW. At the time of this study, no NGO was implementing a project in the rural community unit.

**Density and reciprocity:** We measured density to give a sense of how well health-related information flowed in both social networks. On a scale of 0 to 1, the overall density in the rural community unit was 0.41, while density of the urban slum CHC was 0.76. Density among actors who represented government institutions was 0.57 and 0.85 in the rural and urban slum networks, respectively. This shows that exchange of health-related information was predominantly driven by and more efficient among actors who represented government institutions.

In addition, we measured reciprocity of the network to get insight in the direction of information flow. Reciprocity indicates to what extent the relational ties are two-way or one-way flows of information. The reciprocity in the rural area was 85%, while it was 92% in the urban area. This means that 92% of the ties in the urban area were reciprocal. Though this was slightly higher than the 85% in the rural area, both are quite high, indicating that actors not only provided information, but also received information in return.

**Degrees of centrality:** We measured degree of centrality to establish which actors were central in information flow and exchange within both social networks (Table 4). In both settings, CHEWs were the most central actors, because they exchanged health-related information with all actors in the network. CHVs in the rural network had a higher level of centrality compared to those in the urban slum. CHCs in both settings were comparatively less central in the exchange of health-related information, although the urban CHC had a relatively higher degree of centrality. The officer in charge of the rural health facility received health information from about half of the network, but only shared out information with three other actors in the network. The nurse in charge of the urban health facility shared information with almost all actors in the network. Another observation was that the Ward administrators (administrator in the county government who is responsible for coordinating county activities, including health activities, at Ward level) was involved in the urban, but not in rural CHC’s network. The local chief in the urban slum had higher centrality compared to the chief in the rural CHC.

**Table 4:** Degree of centrality and betweenness in the rural and urban slum CHC’s social network.

	Rural CHC network			Urban slum CHC network		
	Providing information (Out-degree)	Receiving information (In-degree)	Betweenness centrality (Number of times a actor was a bridge for health information flow)	Providing information (Out-degree)	Receiving information (In-degree)	Betweenness centrality (Number of times a actor was a bridge for health information flow)
CHEWs	10	9	34	11	10	7
CHV	8	10	28	4	7	1
Sub County Level Government Health Managers	5	3	9	11	9	3
Local Chief	5	5	3	10	9	3
CHC	4	4	0	7	8	2
Officers in-Charge of local health facilities	3	6	3	10	10	4
Ward administrator	0	0	0	7	8	0

This analysis indicates how isolated the rural CHC was in providing and receiving information related to health activities. The CHC in the urban slum had a slightly higher degree of centrality than the rural CHC, but it was still lower than that of the government actors.

Our findings show that CHCs were actually not the central actors in the exchange of health information within the community unit. Based on the qualitative data we collected, there are three potential reasons to explain this: First, CHC members did not have the required resources and incentives to play their roles effectively. Second, CHC members reported not having the requisite skills to perform their roles, despite their potential to be prominent actors. Third, there were indications from primary health care workers that CHCs were systematically left out in planning and decision making related to community health services, as shown by the following quotes:

*“… but you know we normally have challenges with the resources and facilitation is really a challenge because the County government has not factored in Community Health Strategy”* (Male rural sub-county health manager, SSI)

*“You need to apply some skills which you sometimes don’t have* … *We need to be trained on a lot as CHVs are”* (Female participant, urban slum CHC group discussion)

*“It’s like these CHCs don’t exist*, *are they featured anywhere*? *They are not featured anywhere”* (Female urban slum CHEW, SSI)

**Betweenness centrality:** We defined betweenness as the number of times that an actor in the social network served as a “bridge” between other actors who were not directly connected. As illustrated in Table 4, CHEWs in both networks bridged the highest number of actors. The rural CHC did not bridge any actors while the one in the urban slum bridged two actors (local chief and officer in charge of the local health facility). We observed this low level of betweenness among CHCs despite them being connected to actors that were prominent in the exchange of health information, such as CHEWs and local Chiefs.

**Diameter of the network:** The diameter of a network represents the linear size of the social network, representing how easy the information flows between actors. It is calculated as the shortest distance (average path length) between the two most distant actors. In both the urban and rural network the shortest distance between the most distant actors was equal to 3. However, the average path length was slightly longer in the rural community unit (1.23 versus 1.17). It is notable that CHEWs in both settings had the longest average path lengths in their networks. This implies that information to and from the CHEW goes via other actors more often than this is the case for other actors in the network. Taken together this implies that information flow in the urban area is a bit more efficient than the information flow in the rural area.

## Discussion

Our study sought to contribute to the body of knowledge on “invisible” patterns of information flow within social networks of CHCs in a rural and urban slum in Kenya. Using social network analysis, we were able to identify the key actors who provided and received health-related information to and from CHCs, and how these actors were positioned in the information pathways. Our analysis revealed that actors affiliated to the government and CHVs were comparatively more central in the information pathways than CHCs and that CHCs had a limited number of actors with whom they exchanged health-related information.

By being the “face” of the health administration and national government at the grassroots level, government representatives (CHEWs and local Chiefs) were influential actors because they had control and were in the center of information pathways. As a representative of the national government, local Chiefs are often *ex officio* members of different committees at community level, for example CHCs, Health Facility Management Committees, community policing and other village project committees. In most parts of Kenya, local Chiefs use their authoritative power to convene community health dialogue days and public meetings *(barazas)* [37]. The relatively high degree of centrality of Chiefs, especially in the urban slum, makes them potential allies for CHCs in legitimizing their actions and decisions related to community health and ensuring implementation of policies at grass-root level. CHEWs are central within community unit networks because they are crucial conduits of information between government health managers, NGOs and community actors. CHEWs also wield power because they can select who they want to work with to implement health interventions. In the case of our study, CHEWs preferred working with CHVs. The high (individual) centrality of local Chiefs, CHEWs and CHVs in the rural setting did not assist in increasing the centrality of CHCs, despite them being official CHC members. There is a need to further assess how the high centrality of some CHC members and dual roles of others could place CHCs in a better position of having more power and control over the distribution of material, informational and health resources in their networks [38, 39]. Kenya’s community health strategy envisions collaboration between CHCs and health facility management committees. However, in our study areas, health-related information and resources were not exchanged between these committees. It seemed that health facility actors (officers in charge and the health facility management committee) did not recognize CHCs as important actors for information sharing and decision-making. This finding is supported by Omeara *et al*. (2011) who documented how community level governance committees were not involved in decision-making by primary health care workers in a coastal region of Kenya [40]. The CHCs in our study also missed out on opportunities for advocating for the community health agenda and allocation of budgets, because they did not interact with members of county assemblies, who are grass-root level representatives in the county parliaments.

Social influence is a major factor that determines how information flows within a social network. According to the social influence theory, as postulated by Lazarsfeld *et al*. (1944), informal interactions between actors in social networks is a powerful mode of transmitting information and influencing attitudes and behaviors [41, 42]. The influential and central actors in the flow of information within a network are called opinion leaders. In the study of social influence, opinion leadership is the degree to which actors in a social network are able to influence others through inconspicuous interactions. Their central position in the networks gives them access to many other actors in their social network. Opinion leaders are also trusted sources of information with the ability and skills to share health-related information with others. Although community members and government health managers perceived CHCs (as entities) to be opinion leaders, our study demonstrated that this role was not fulfilled in practice. Instead, CHEWs, who represent government health departments, were the opinion leaders in both community units.

An exploration of 581 meeting minutes from 129 Nigerian CHCs found that CHCs functioned in different but inter-connected modes. These modes were: bridges between community and government (*community connectors*); agents of social accountability (*government botherers*); entities that oversee the day-to-day running of primary health activities (*general overseers*); providers of a forum for discussing primary health challenges and solutions (*village square*), and; some functioned as a back up to the government in addressing primary health care challenges (*back up government*) [10]. The functions of CHCs as identified by this Nigerian study can be identified as CHC functions in the Kenyan Community Health Strategy as well. CHCs in our study functioned as community connectors/village squares, to a limited extent, by working with the local Chiefs to convene quarterly dialogues days. It is during these forums that community members interacted with government health workers and shared their health care priorities. CHCs in our study also functioned as “back up government” by supporting local Chiefs to implement government directives, such as compulsory child immunization and discouraging banned cultural practices.

The peripheral position of CHCs in both social networks meant that they were not able to participate in the exchange of information and decision-making on community health services. This contributed to demotivation among CHC members. Possible actions to motivate CHCs would be to enhance the capacity of CHCs, providing them with operational support and formal recognition that would strengthen them to be more central actors in their community units. It has been documented elsewhere that building skills of community level health workers was a great source of motivation and retention [43–45]. Kilewo *et al*. (2015) also found that lack of resources to facilitate their work and training in managerial skills was a major barrier for effectiveness and motivation of community based committees in Tanzania [46]. Another intervention to motivate CHC members would be to institutionalize CHCs as part of the county government and providing both financial and non-financial incentives such as recognition awards and transport reimbursements. As witnessed in the Brazilian Family Health Programme, this approach can succeed in motivating community-based committees to play their governance and oversight roles. Such a policy change would place community participation through CHCs as an integral part of county governments’ responsibility in delivering primary health care, which has been shown to improve self-efficacy of health committees [47, 48]. Our study findings support World Vision International in calling for Ministries of Health to put in place important programmatic, structural and policy elements for CHCs to function effectively [49].

Our study had limitations that should be factored in while interpreting our study findings. We conducted this study during an electioneering period and some of the persons who had been named as actors could not be reached due to involvement in either managing security or in political campaigns. To compensate for this limitation, a second set of interviews was conducted to explore the contacts of the persons who could not be reached. In one community unit, the CHEW was interviewed to confirm the network of the local Chief and chair of the local community policing committee. This may have introduced bias, because the CHEW responded from her own perspective. Secondly, we relied on the County Health Department to select a study site and on the CHEWs to sample community members to participate in the FGDs. FGDs with the CHC and community members in the urban slum setting had a lower turnout of participants than expected. The lower turnout was because several of the invited participants were casual laborers and small business owners who were not able to get time off from their occupations for an FGD that we organized on a weekday. This possibly resulted in a selection bias. Third, we did not analyze the influence of trust, shared values and power dynamics in the CHC networks. Analysis of individuals’ power and influence may have revealed whether power dynamics within the community may have hidden effects on how actors in these networks make decisions, irrespective of their position in the network. Finally, our case study was conducted in two community units, and findings are not generalizable with other contexts. However, we believe that the issues we documented cut across many of Kenyan community contexts, and further research is needed to confirm this.

## Conclusions

According to the Kenyan Community Health Strategy, CHCs are expected to be central actors in their social networks. By being a central actor they could represent community members in leadership and oversight in delivery of community health services, but we found CHCs currently do not fulfill this role. Counties may need to strengthen CHCs to play their role at the heart of community health service delivery, by building their capacity in advocacy and governance, recognizing the role that they play in community health services, and providing basic financial incentives and operational support (e.g. transport and meeting allowances). The Community Health Strategy gives CHCs a central role in community participation in overseeing community health services. There is need for decision makers to explore ways to better use double roles of CHC members to allow better inclusion of CHCs in health-related information flows.

## Supporting information

S1 File S1_File. (XLSX)

S2 File S2_File. (XLSX)

S1 Table S1_Table. (DOCX)
