# Supplementary material for: “It’s like these CHCs don’t exist, are they featured anywhere?”: Social network analysis of community health committees in a rural and urban setting in Kenya
Source: PLoS One. 2019 Aug 8;14(8):e0220836. doi: 10.1371/journal.pone.0220836 (PMC6687128; doi:10.1371/journal.pone.0220836)
Supplement: S1 Table — (DOCX) [file pone.0220836.s003.docx]

**Themes**

1. CHC factors
2. Health facility factors
3. Health system factors
4. Societal factors
5. Community factors
6. Functionality factors

**Summary based on charting the themes**

1. Community members perceived CHCs as important in community health. CHC members comprise opinion leaders in the community and are chosen due to the roles they play in the community
2. CHVs are the center of the network in the community units. Counties, NGOs and CHEWs passed health information to CHVs and by passed CHCs.
3. CHC did not share health related information with the local Health Facility Management Committees, HFMCs did not share any information regarding decisions on primary health and on resource allocation
4. Despite feeling being left out by other actors in their communities, CHCs often worked with local Chiefs to implement government directives e.g. anti FGM, compulsory immunization
5. County governments has not provided resources for community health services. This affects support to CHCs
6. CHCs are well respected members of their community and they use this role to mobilize communities

|  | **CHC factors** | **Health facility factors** | **Health system factors** | **Societal factors** | **Community factors** | **Functionality factors** |
| --- | --- | --- | --- | --- | --- | --- |
| ***Charting*** | *Community health managers prefer working with the CHEW as the central actors for relaying health information to the CHVs. CHCs are not in the information network* | *HFMC has not involved CHC in making plans for the health centre. CHCs are not represented in the HFMC. CHCs stated they were left out in sharing these information* | *CHEW indicates minor role that CHCs are engaged in the health system plans*  *Senior managers did not have any connection / networks with CHCs. The Medical Officer did not have information about the CHCs* | *Respondents stated that devolution of health services from national level to Counties had a negative effect on the position of CHCs in their networks. Counties do not fund community health. This therefore weakened the role of CHCs* | *CHC members are well respected by the community. Despite having other leadership roles in the community, CHC members did not play a central role in the oversight of community health communities* | *It emerged that CHCs in our study did not meet regularly due to de motivation because they felt left out on the community unit networks. They reported being relegated to the periphery of the community health network.* |
| **Rural County CHS Coordinator** |  |  | Res: I think most of the community health agenda is partner driven. We have no direct resources that go direct from the government to the community units. We find it easier engaging the volunteers more and trying to tend to…Because of the results; immediate results and overseeing the CHC role because there are no immediate results that you get by engaging them. |  |  |  |
|  |  |  | They are never featured so you ask the new CHEW do you know CHC member and they are like I know the CHV so the CHVS have kind of overshadowed the CHC and they are not really seeing their role meet maybe because it’s not us who are facilitating the meeting this is a CHC, he will be coming for your meetings |  |  |  |
| **Rural SC- MoH** | Int: so who in the community is responsible for this community policing, who from the community participates?  Res: Well, the CHEW for us is key and through the CHEW we also involve the CHVs and in some of the places where we are… because in the Sub- County; the whole Sub-County we have only 7 CHEWs | Res: So we have to make the best with the drugs we have. Because I also realized the process of… there have been occasions where the community have accused the health workers of not being optimal in their service delivery and it has been because of issues of lack of transparency that you are not able to help them but you don’t make it known to them it is beyond you. | Int: then there is another one that is usually more concerned with community strategy. So maybe I can even start by saying, how active are they in Kajiado South?  Res: That is a question that can be answered by Samson the (… 16:35 not clear) honestly because he is my focal person. Will you address the same thing to him?  Int: Yeah. So I will pick different points from the different pages, but that one I can focus…………..  Res: Yeah at this point, at that point, Samson has more idea on that. |  | . They are the people the community listens to and everyone loves to be valued, everyone loves their opinion to be valued and love to feel important so through these CHCs, they feel that they have a voice | Res: Being consistent in execution of these planned out activities would really be key. Then leadership; having the right leadership here, fine, but we also need support from our leaders. Both above and at the same level, we need to speak one language |
| **Rural SC – CHS Coordinator** | ... I was telling you from the beginning that age is always a factor in terms of addressing any issue in the community. You know in Masai land the culture itself speaks for itself and you cannot as a young boy you cannot come and start telling me that we should do this this; this; NO. | Int: do they ever meet or how do... do they have any form of relationship?  Res: I will say, No.  Int: Ehe  Res: because what normally happens you know if let’s say for example maybe what will happen if one of the CHC member has been selected to be part of the committee and the advantage that most of the time we have is that the chief is normally let’s say if we have an area where we have a chief who is very close to the unit and very close to the facility I say they will represent us in the Health Facility Committee but it has been quite a challenge because we are trying, that has not really strengthened the linkage between community health unit and the facility. | Res: for community health services. I also Coordinate community dialogue days and implement such activities and community action days and also the other thing that I also do, I also most of the times access, I also evaluate the performance of this community Health units though I have quite a number because in Kajiado South we have 24 Community Health units  Res: but you know we normally have challenges with the resources and facilitation is really a challenge because the County government has not factored in Community Health Strategy. We don’t have money that we will use to implement these activities | ... I was telling you from the beginning that age is always a factor in terms of addressing any issue in the community. You know in Masai land the culture itself speaks for itself and you cannot as a young boy you cannot come and start telling me that we should do this this; this; NO. | Res: CHCs, I will say in this community we have a challenge with cultural beliefs and also the CHCs as I was saying in most of our CHCs we have chiefs and there is challenge and you know out of those cultural beliefs and cultural practices we have Female Genital Mutilation and while we are having that... which has really been a challenge because our CHCs are doing a lot when it comes to the time to fight those vices | Res: what they normally do is that you know our Community Health Volunteers and the CHCs also come on board, they have monthly meetings and let’s say when it gets to the time that we are doing a Bi- annual or an annual work plan, they will sit together and develop like what kind of activities do we want to do but you know the challenge has been you know most of the activities you plan but they are not implemented because there are no resources. So that is actually not really boosting the morale and that is why we are having even a lot of CHC, even CHV attrition. Yeah |
| **CHEW Rural CU, Rural South** | facility they are not involved and they don’t involve themselves because there is that health committee for the health facility | …are they involved in hospital plans in the health centre?  Res: they are not involved. | the CHCs are involved but not very much. Let me say not that much. We plan with them when CHA has an activity he/she involves them but also its one of them; to plan is their work but they do not so much |  | Yes. You know they are also leaders and also you can find somebody else is a chairperson in a school, he/she is managing the school; he/she has been chosen by the community… | They have helped so that the services can be completely accepted and also they are heard; they are heard for instance on latrine issues; if a health professional goes round and find an area with no latrine in the community you just inform the CHC. |
|  | *CHCs helped to foster unity and motivation among CHVs. CHEW believes that may reduce attrition of CHVs* |  | *CHCs did not play a central role in developing community health work plans* |  |  |  |
|  | Res: on the achievement is unity because, I have told you we are 32 CHVs even if not all. the CHCs have been able to unite those CHVs with the community at large because you know CHVs are volunteers and they are the ones who do a lot of work so the CHCs cam motivate them by telling them to go on with that work, to work and they don’t leave. |  | if its work plans maybe I sit down and plan; we plan with them if there are activities but not very much it’s just a little bit because we meet with them quarterly. Mostly we meet with that committee 3 times; after three months we meet sometimes when there is a demand we meet monthly. You find maybe if something happens at the middle so they are not very much involved. They are not involved mostly |  |  |  |
| **Rural South CHS Coordinator** |  |  | *Community health services are underfunded. This affects capacity building, supervision of CHCs* |  | *Community practices that CHCs help to address together with the village chief. They are however not well facilitated* |  |
|  |  |  | Res: but you know we normally have challenges with the resources and facilitation is really a challenge because the County government has not factored in Community Health Strategy. We don’t have money that we will use to implement these activities | so and most of the other things that I am trying to you know in Masai land, you know I am a Masai personally so I know so many things that happen behind the rules | …CHCs, I will say in this community we have a challenge with cultural beliefs.. and also the CHCs as I was saying in most of our CHCs we have chiefs and there is challenge and you know out of those cultural beliefs and cultural practices we have Female Genital Mutilation and while we are having that... which has really been a challenge because our CHCs are doing a lot when it comes to the time to fight those vices and other things and also resources; resource is also quite a challenge |  |
|  |  |  | *CHCs are viewed as the gatekeepers in their communities* | *Leaders are generally trusted in the Maa community. Being leaders, CHCs are trusted by the community based on their leadership rooles* | *The health system manager believes that communities should be given a “voice” through CHCs* |  |
| **Rural South MoH** |  |  | Res: Yes, so if you have matters health you need to pass across to them, it is very important that you have the chiefs, village elders and even some of these politicians on your side. You cannot avoid that. So through the same people that is how we communicate to the community, | There is a lot of trust with the local leaders, the chiefs, village elders a lot, and not maybe in Rural south but in the Maasai community. | They are the people the community listens to and everyone loves to be valued, everyone loves their opinion to be valued and love to feel important so through these CHCs, they feel that they have a voice. A voice that is spoken, a voice that is heard as far as you know implementation or development of any policies and guidelines, so we can just look at |  |
| **CHC RuralFGD** | *CHC members are well respected in their communities* | *CHC members lack training. Last training was facilitated by an NGO* | *CHCs only get recognized when there is a problem. This may be a fault finding approach from the health system managers* | *CHC members have to use their own resources to serve community members* | *CHC implement health related policy directives in the community* | *CHC also have some administrative duties* |
|  | Male respondent 4: You know a CHC is someone who is recognized by the community and is respected by the community. | Male respondent 3 : Now after one year , we formed the CHC. The ones who were initially selected got trained by AMREF but the current CHCs have not received any form of training. | Female Respondent 5: Ok and when their work looks good they are fine but when things are not working that’s why they will come down complaining why things are not going well | Female respondent 2: Ok as we are still on CHCs we have challenges like now there is a strike, people bring their challenges to us so that we can resolve issues but there are no funds, so what we do is we go back to our pockets to assist the community members   - - - - - - - -   Male respondent 4: Some times when we need to mobilize people for meetings the pockets have drought and you can’t walk to invite people so you need to call and you may not have credit  Female Respondent 5: For example, a mother is here she is ready to deliver and she has no transport money, you are forced to go back to your pocket and provide for her | Female respondent 2: Like there is a time we had a measles outbreak, the child is not able to be brought to the hospital because of religious stands, we are forced to steal that child and bring her to hospital …. | Female respondent 2: Like when there is a CHV who has dropped out we organize with the community for meeting with the CHC where we select a person of their choice to replace the CHV. |
|  | *The CHC is made up of persons well recognized in the community* | *Facility HC side-lines the CHC in sharing resources* | *Health system support for CHC is absent* |  | *CHCs can mobilize community due to their prominence in the community* | *Administrative duties played by CHCs* |
|  | Male respondent 3: Another thing is that the CHCs are not only limited to working on health matters we are involved in others ways for instance I also work at the chief’s office, like this mzee is in charge of nyumba Kumi, I mean we have to be people who are trusted in the community.  Male respondent 4: The community recognizes CHC is when something has occurred abruptly or when we are required to mobilize the community that’s when they recognize you because you are their leader | Male respondent 3:And at times this committee is the one that distribute the land that belongs to the facility, so we have ever told them that the CHVs and CHCs should be given at least an hectare to cultivate and put the money earned into the group account as savings, so that this can be a motivator , but they said they will give us as CHVs and CHCs but we have never received from the HOD but it ended up being like a personal thing people giving it to their relatives and the land in this facility is big its 10 hectares | Female respondent 2:That’s why am saying if we had partners and the government also to be able to recognize such people we would make much progress and we would be able to achieve the percentages , even if it’s not 100% but 99% |  | Male respondent 3: These are respected people when they call for a meeting people attend, if it’s the CHVs they attend meetings when we call, if it’s the water people they come and we are able to address them. | Female Respondent 6: You will find that they are not able to be confidential therefore they have to be removed and replaced. |
|  | *CHCs not trained in their roles* | *Deteriorated relationship between the CHC and HFMC* | *No investment from Health system to train CHCs* |  | *Community understands them* | *Need for training* |
|  | Female respondent 2: 1^st^ thing is we know a lot about leadership, from what we have shared you realize we are leaders. Currently people are digital and when we go to the community we need to be able to have some form of identification .The other thing is that we are not recognized by the government the only recognition we get is in the work that we do but even there we have challenges but we have not reached that section of challenges yet….. | Male respondent 3: There is a time we had a good relationship and there is a time we were asking ourselves why the CHVs do not get support from the facility. There is a time the facility committee had agreed on the 25%, we waited and it was not implemented… do you remember?  ---------------------------  Male respondent 4 : But there is another thing they meet once a month so if they had us they would be able to learn about the issues of the community members during their meetings so that if there is something that needs to be rectified they would do it because they are aware of it . | Female respondent 2: That’s what am saying that from there we don’t know the way forward from there we have not received training on proposals that can help us make progress and the learn ways of following up with the county and sub county so that we can learn how we can partner with them so that is what I have told you that we need to be trained so that we can know what our duty is and our work because all these things have been brought about by the government but we have not known what is the way forward. |  | Female Respondent5You will find that in that community they are understandable and when they speak the people in the community understand them. When it comes to school matters the people know that these are the people who help us even with school matters, like for example when there is a health issue in the Maasai community, when we go there and speak to them they understand us. For example like right now we have this meeting, you will find people wondering, who is that person and where is he from, but when we are sited here like this, we understand what | So when people are trained the people can see that this person is a leader. So it is very important for CHCs to receive training on leadership. |
|  |  | Male respondent 3:And at times this committee is the one that distribute the land that belongs to the facility, so we have ever told them that the CHVs and CHCs should be given at least an hectare to cultivate and put the money earned into the group account as savings, so that this can be a motivator , but they said they will give us as CHVs and CHCs but we have never received from the HOD but it ended up being like a personal thing people giving it to their relatives and the land in this facility is big its 10 hectares | Female respondent 2:That’s why am saying if we had partners and the government also to be able to recognize such people we would make much progress and we would be able to achieve the percentages , even if it’s not 100% but 99% |  |  | the 1st lot was trained and some have dropped, so for this lot here I don’t think there is any that has been trained CHCs, am speaking for the current CHCs we have. I am the only one who is lucky to have been trained because I was part of the 1st CHC lot and I have been lucky to be selected again to be part of this CHC, but for this group no one had been trained as CHC. |
|  | *The CHEWs and Chiefs nominate community members to serve as CHC and ask other community members to approve during dialogue days* | *Importance of CHC,s gatekeeping role in the community* | *Recognition by NGOs and county health managers is a Source of motivation for CHCs* | *Chiefs are powerful persons in the community and CHCs often engage them to validate their messages during dialogue days* | *Recognition by other actors in the organization is a source of motivation for CHCs* | *CHCs reported that CHVs are often taken for training and they are not involved* |
|  | Male respondent 4: For CHC we were selected in a meeting, the chief will be present the CHEW will be present, and the CHCs will also be there. Like MIORA , they sat and decided to have a meeting , the invited the chief, CHEW and I think there were doctors/health officers. The senior doctor was present and the doctors in charge of the community and there now they present the CHV and they say this person is fit to be a *community doctor.* | Female Respondent 5: It would be good because we are people from the village and we have information regarding the village, so if they involved us we would contribute and bring information back and they will ask us regarding the problems we have identified on the ground and what are our recommendations in order for the situations to improve. | Female respondent 2: You know when you are recognized by an organization or by the government, you are encouraged because you know that there is someone who is aware of your presence on the ground and recognize the work you do in the community so that even though you don’t get a big kind of support but the community is proud that they have a leader among them who can champion their issues | Male respondent 4: But when we are having these activities like dialogue and action day we have to involve the chief and the chief also must attend. We involve the chief | Female respondent 2: You know the people who really recognize us are the CHVs, the community does not recognize us much because, you have seen we 1^st^ came in as leaders then as CHC, now where we are we receive reports from the CHVs we, we sit discuss and decide on the way forward but if you talk about the community recognizing us it will be difficult because all they know is *community doctors* | Female respondent 2: What I was saying is the 1^st^ lot received training but the once that were recently selected have not received any skills on how to deal with people but they are applying their CHV skills in their CHC work |
|  |  | Male respondent 4: Ok when the facility committee was selected it would have been nice to have a CHV or CHC representative at the committee but now they are the leaders. However there is a CHV who is part of that committee thought it’s for a different purpose, a role he place on the ground he is the chairperson for the water project that’s why he is there although now we would like a chance though is also a CHC from our community. But we have not been given a chance | Male respondent 3:The challenge that we have is mainly poverty, it has contributed so much to CHV attrition because of their lack of income. What I would like the government to intervene on this matter. The only help we have received are the equipment that AMREF gave us the services that we can offer are written there, photo copy, browsing, scanning, video show, computer services, sending fax printing and such like things  Female Respondent 6: They should recognize us even when work is not running smoothly in order for the work to run smoothly, that can help us do our work very well  Female Respondent 6: Because if they fail to recognize the work we do then even at the community level we will not be recognized we will not be respected at the facility but if they boost us as county government then we will be important people who are respected. |  |  |  |
|  | 1. *CHC members in the urban settings reported that lack of financial incentives was a primary reason to demotivation. They ended up dropping out of the committees* 2. *Lack of motivation led to shirking of duties by CHC members* | CHCS reported that they are never engaged by the health facility committees in any activities. | CHVs receive more attention from actors in the health system. They have more access to health related information and have access to more health system actors |  |  | *This primary health care worker believed that lack of sufficient training, stipend are a major contributor to the demotivation of CHCs* |
| Urban Community Health Coordinator | for one they really feel left out because even when partners come they have really equipped the CHVs they have done so much training with the CHVs yet the CHCs know nothing, when there are stipends, there are no stipends for CHCs stipends are all for CHVs they feel that no one remembers them, they feel that people remember them when things are not good but when things are good they are left out you know they say when a partner comes to introduce a project they have to do a presentation to them so they feel that when you open the gate you forget the gate keeper because you come in you implement and you forget us you don’t even tell us when the project is coming to a halt you don’ t call us when you are reviewing whatever you are reviewing they completely feel left out  R: let me say the Nairobi ones is like they are doubling in as CHVs if they were not doubling in there would be no connection especially in Nairobi, just because they double in I think they feel more motivated | presentation to them so they feel that when you open the gate you forget the gate keeper because you come in you implement and you forget us you don’t even tell us when the project is coming to a halt you don’ t call us when you are reviewing whatever you are reviewing they completely feel left out | for one they really feel left out because even when partners come they have really equipped the CHVs they have done so much training with the CHVs yet the CHCs know nothing, when there are stipends, there are no stipends for CHCs stipends are all for CHVs they feel that no one remembers them, they feel that people remember them when things are not good but when things are good they are left out  R: they are not the ones who do it is with the help of the CHEW because how are they going to do a work plan and they don’t understand those things, they are all health indicators they know nothing about so you are telling them to plan for what unless they plan for the dialogue days  : that one they are not yet there you know mostly trainings they are left out so you are talking about family planning they don’t know what family planning is to them it does not add up unless you’re a CHV and mostly the ones that double in they are not that powerful you know like the village elder | It’s all about power and ownership you know in anything you need the political good will and Nairobi being an urban set up the powerful people are the landlords you might make a declaration let’s do this the landlord might decide nobody will enter my premises and there is nothing you can do so for you to get that political good will they must understand the work a CHV does they also do the work a CHV does and funny enough most of them are the ones you will find around because they are not going to work because they have an income from the rent so they are readily available | so if you start doing something with the tenants and you are not involving the landlords you will not go anywhere so what we have done in Nairobi and it is working per se they double up because the way community strategy is centered there is nothing much for the CHCs so even when there are trainings and there are stipends there are for the CHVs because you can imagine if your tenants are getting the stipends and you as the landlord not getting anything you’ve mad them to double up and it is working for us  R: yeah it’s very practical if you do not do that you will not do anything, you will not work they will not allow you to do any work there because they own the community and you are not talking their children to be CHVs unlike the rural set up | for one they really feel left out because even when partners come they have really equipped the CHVs they have done so much training with the CHVs yet the CHCs know nothing, when there are stipends, there are no stipends for CHCs stipends are all for CHVs they feel that no one remembers them, they feel that people remember them when things are not good but when things are good they are left out  ------------------------------------for one they really feel left out because even when partners come they have really equipped the CHVs they have done so much training with the CHVs yet the CHCs know nothing, when there are stipends, there are no stipends for CHCs stipends are all for CHVs they feel that no one remembers them, they feel that people remember them when things are not good but when things are good they are left out you know they say when a partner comes to introduce a project they have to do a presentation to them so they feel that when you open the gate you forget the gate keeper because you come in you implement and you forget us you don’t even tell us when the project is coming to a halt you don’ t call us when you are reviewing whatever you are reviewing they completely feel left out |
|  | R: that is unavoidable in Nairobi because Nairobi being an urban set up it is different from the rural where in the rural are the owners and their children might become the CHVs now in Nairobi if you leave out the CHVs to become the CHCs you will not penetrate because the CHCs are normally the landlords they are the owners so if you start doing something with the tenants and you are not involving the landlords you will not go anywhere so what we have done in Nairobi and it is working per se they double up because the way community strategy is centered there is nothing much for the CHCs so even when there are trainings and there are stipends there are for the CHVs because you can imagine if your tenants are getting the stipends and you as the landlord not getting anything you’ve made them to double up and it is working for us |  | M: and finally what keeps the CHCs motivated?  R: let me say the Nairobi ones is like they are doubling in as CHVs if they were not doubling in there would be no connection especially in Nairobi, just because they double in I think they feel more motivated  M: yeah  R: and they feel like I’m the one passing this information about health, I’m involved you know my people are seeing me delivering services and you know a CHV in Nairobi is called a doctor that title doctor really motivates them |  |  |  |
|  | Unfortunately the reports I’m getting is that since last week when I heard about CHCs; that is when we began to talk about them, is that there used to be CHCs but at some point the function of the CHCs and the functions of the CHVs was not very clearly demarcated; some of the members of CHC were also CHVs. Because these roles were not very clear and it seems like the CHVs have a lot of activities and being a CHV seems to be a little bit more rewarding than being a CHC, you find that people who were initially CHCs and could easily transform into CHVs just became the CHV and ignored the CHC function. |  |  |  |  |  |
| CHC FGD | **Male Respondent 1**: We can give you; since we started out unit up to date, there was a time it went down a bit because there was no motivation. You know when there is no motivation; women are going to work, everyone is going to hustle; we came to Nairobi to hustle not sit. You can’t decide just because you are volunteer, so you time should be wasted all the time. There was no motivation but we have revived it and decided to do it; God is there.  ______________________  Int: How have they performed in providing that oversight in their respective community units?  Res: Not very well. These people are not motivated. You will call for meetings; they will not be there because they are not motivated; this is Kibera. I wouldn’t say they have performed so very well. If I would rate them, maybe about 40%. | Mod: There are usually two committees; the facility committee called Health Facility Management Committee, and then there is the CHC. Do you have any relationship with that committee?  Male Respondent 1: We have a relationship…  Mod: At the hospital or let’s begin, does it exist?  Male Respondent 1: It exists but it has gone down because when we started, for the first 4-5 years, we could have meetings of the health facility; apart from the one of CHC. We could meet because as CHC chairperson is a member of that committee. | Female Respondent 2: Another thing, more trainings are being conducted on CHVs. So also CHCs should be brought more training because they are the ones supervising these ones on the ground and they are the ones who lead them. If all the time it is the CHV who is being trained, it becomes another challenge because the CHC also needs that training. You can’t approach a student; you are the teacher but the student knows much more than you. How will that work? You will find a CHV when training like disability comes; it is the CHVs who want to be trained. Whereas there is a CHC on the ground who doesn’t know about these training on disability; what should be done. The CHC should also know so that when he/she finds that a CHV hasn’t done the necessary in his/her household which has disability, he/she can react, going to supervise. | Mod: Do you deal with politicians?  Res: No, we don’t deal with politics.  Mod: Like proposals?  Res: None; where can they be found?  Res: If the politician is down?  Mod: They don’t support you?  Res: Even if you write for them business plan. | **Female respondent 1**: we sat down in a gathering and in that gathering we selected each other after selection the chief told us our job description and how we were going to help each other but after that day he told us there was something that was to come to benefit us from that day we have never received any assistance we used to be told the government is looking after us and it will be paying us from 2011 till date the government hasn’t looked after us | dialogue and even the chief attends the dialogue  M: are they the same ones who do the action day also  R: yes, we mobilize for action days you know you are now the one in the community and in the community it’s a bit tricky you can’t just start doing things especially in an urban center having some meeting and the chief is not aware or the village elder who is the nyumba kumi person so they help us |
| CHEW 3 | Res: Unlike the CHVs, there are many trainings they undergo and the CHCs are supposed to oversee the CHVs. You find that these CHVs have more knowledge than CHCs. Everybody comes, the training comes and they go for training and the CHCs are nowhere to be seen; they are nowhere to be trained. There is nothing they are gaining. |  | Res: What we have. We can’t form other new CHCs, can’t form other committees without partners, without resources; it is very tricky. I have always worked with what I had at that particular moment. | Int: How do you say devolution has affected governance at community level; by that I mean CHCs?  Res: Very much because they don’t seem to exist. As much as devolution took place up there, us we haven’t witnessed the devolution yet. It’s like these CHCs don’t exist; are they featured anywhere? They are not featured anywhere. | It’s tricky. For us we know amongst ourselves, but how will that person be known; where. In return he doesn’t have services that will…The only part that he/she might be recognized is in a *“baraza”*, of which those councils are so limited. When you call him to come for the council, and you don’t give him anything, you think he will come? If the chief himself is part of the CHC, when he is called, he doesn’t just come… | Res: Not very well. These people are not motivated. You will call for meetings; they will not be there because they are not motivated; this is Kibera. I wouldn’t say they have performed so very well. If I would rate them, maybe about 40%. |
| CHEW 1 | Very much because they don’t seem to exist. As much as devolution took place up there, us we haven’t witnessed the devolution yet. It’s like these CHCs don’t exist; are they featured anywhere? They are not future anywhere. |  | I don’t think if I have a CHC. They are there by name but not by action. Most of them are there by name; you can say this and this, but these people are not available. | R: devolution affects CHCs because earlier that structure was very clear but when it came to devolution nowhere is CHC very well felt, it is kind of being silenced |  | Res: When it…The CHC is comprised of how many people, 9 people. The fortunate part of it or the unfortunate part of it is the secretary to the CHC is the CHEW. You will find very many are the times that even when you call upon these CHCs they will never turn up. You end up doing as the CHEW and maybe some of the CHVs on the ground will end up doing the AOPs. |
| CHEW 2 | R: they system is working although they are not motivated and we feel that when we work together we motivate them and we don’t work from distance we work very closely to them | Int: How does the CHC connect with the HFMC; how do they work?  Res: I will still come back; because I don’t have CHCs, I still stand as the CHC who link to the Health Facility Committee. By the | Res: They don’t have stipends, they are not considered when there are trainings they are never involved in the trainings. Once they were trained, it was the first time and the last time they were ever trained. | Where are we supposed to get that help; how has the devolution really helped us or helped the CHCs? These people have been forgotten; they don’t even exist. I’m imagining I’m going to the chief; he’s is part of the CHC | The new ones haven’t been known very much in the community, but they are known because they weren’t from outside; they were just picked among the CHVs who were active. |  |
| **Urban Health facility in charge** |  | We realized that there were gaps CHC’s and CHV’s because every partner that comes asks for CHV’s we’ve realized that CHCs are left although they have their roles and responsibilities in the community because in the facility committee the CHV’s maybe 2 representatives have to be there and a CHEW who is a secretary of it the but when we came to realize that this committee is very important to the community because there is no entry to the CHVs without reaching them CHCs, do you get me. | R: the community right now has challenges because the budget is not trickling down to the communities, that’s the challenge that we are having we did our work plan and it had its budget but it hasn’t reached to the community as of now | R: yes that’s a gap definitely because anyway you know health has no political milestone | R: yeah because you know leadership is so diverse I would say there was a gap because what you get from the CHWs tells you there us a disconnect between the CHCS and the CHVS because some of them are not willing to share their issues with the CHC members but you know those ones come because of the differences that might have existed prior to us engaging them and you know as human beings we don’t tend to forget | R: their challenges one of them is that they are not facilitated monetary because they are purely doing voluntary work number 2 I can say even the training that they were given the timing itself was quite limited so they need like refresher on leadership so that they are able to handle more complex cases in the community. |
| **FGDs with Community members** | | | | | |  |
| *Community perception of the importance of CHC* | | | | | | |
| Mod: OKAY; thank you. You mentioned about dialogue day; what exactly happens?  Res: Before they call for it, they usually announce the dialogue day. All the community members; from *[village names omitted]* and *[village names omitted]* we attend. They educate us on how they are getting on and the step they have taken. We feel that they have helped us. | | | | | | |
| Res: On my side, the committee has been of great help because in case of an emergency issue, they come to the village and educate people; they call for dialogues, we are called for those meetings and they let us know. Let us say they have been our eyes here in this community. If you get any problems, even when you are hungry; they struggle very much to assist those who don’t have strength. | | | | | | |
| Res: If I may add, there are community doctors who work together with the committee. They have each been given households to serve and they usually have meetings. Those who have been chosen to make that committee, usually supervises how they work. | | | | | | |
| Res: I feel the committee is good because they work hand in hand with CHVs and when there is a problem, they assist the community. There is a period we see them moving around with dewormers which they give people. They also teach us that we know how to give animals medicine but we don’t know how to give dewormers. We know how to deworm cows and goats but we forget about ourselves. They assist us in educating us so that we don’t forget about ourselves; they tell us that our health is our responsibility. It is all about one taking care of himself/herself; therefore they have really assisted us. Also we mentioned about the religious group that doesn’t go to the hospital; we have had so many issues here. Where I preach, you find so many people… But after the coming of the committee and the CHVs, things have been going very well; they go and educate them even in their homes, and we see things have continued to change and the fear of that religious group is reducing. | | | | | | |
| Res: On my side in *[village names omitted]* they are the eyes of the community and also our doctors here. For instance on the side of toilets they have been of great assistance. If they find one who is not able, they call community members and we join hands in helping build the toilet. The committee members have also been helpful; there is a certain religious group here who don’t go to hospital. There was an outbreak and they joined hands with the CHEW, doctors and the office of the chief to take the children and give them immunization. They don’t go to the hospitals so they try to hide their children; they are of help because they take those children and give them immunization; so they are doing a very good job. | | | | | | |
| The committee usually follows people up to ensure that; the CHVs encourage people that they should deliver in hospitals. So they insist on that and ensure that they have come. | | | | | | |
| *CHC effect government directives* | | | | | | |
| Res: In [***village name***] they assisted in building toilets; there are no households without toilets in *[village names omitted]*. Also about female circumcision, it’s like there is another government in the village; if you circumcise a child and hide her, it will still be known | | | | | | |
| The community doctor can send a person to hospital and the person refuses, but if they accompany the CHV, things become easy. If they have refused to build a toilet, when the committee and the CHVs join hands, things become OKAY | | | | | | |
|  | | | | | | |
| *CHCs have to use their own resources to serve* | | | | | | |
| Sometimes they enter a household where there is hunger. If they have gone with issues of health and they see those people in such a state, they can’t leave them like that. They go into their pockets and buy them food. When these two people join hands, they face a lot of challenges for sure and the government doesn’t give them anything | | | | | | |
| *Greater recognition for CHVs by community* | | | | | | |
| Res: So when you rebel. The person who has carried; we can say as a community, the one who has carried a huge burden, is the CHV. Because he has to come to me and ask me questions concerning my health and if there is something I have refused to do, ten he can report it to the CHC. So they work together but the person I feel has carried a heavy load is this one. Because sometimes when they come, we rebuke them; that they are lying to us, how come they work daily for free? But they never get discouraged; they are amazing people; even when you rebuke them, they just come again. They are people who are doing a very good job. We also thank God because they have brought about changes; as a community we have seen so many changes that have passed through these people. | | | | | | |
